# How to Verify and Manage the Translational Plagiarism?

**DOI:** 10.3889/oamjms.2016.070

**Published:** 2016-06-24

**Authors:** Viroj Wiwanitkit

**Affiliations:** Suvannhabhumi Clinical Training, Research and Development Center, Institute of Natural Medicine Science Development and Establishment Project, Surindra Rajabhat University, Surin Province, Thailand

**Keywords:** translational plagiarism, Google Translate tool, non-English reference, non-English article, publication ethics

## Abstract

The use of Google translator as a tool for determining translational plagiarism is a big challenge. As noted, plagiarism of the original papers written in Macedonian and translated into other languages can be verified after computerised translation in other languages. Attempts to screen the translational plagiarism should be supported. The use of Google Translate tool might be helpful. Special focus should be on any non-English reference that might be the source of plagiarised material and non-English article that might translate from an original English article, which cannot be detected by simple plagiarism screening tool. It is a hard job for any journal to detect the complex translational plagiarism but the harder job might be how to effectively manage the case.

The recent article “How to Verify Plagiarism of the Paper Written in Macedonian and Translated in Foreign Language?” is quite interesting [1]. The use of Google translator as a tool for determining translational plagiarism is a big challenge. As noted, “Plagiarism of the original papers written in Macedonian and translated into other languages can be verified after computerised translation in other languages [1].” In fact, it is no doubt that translational plagiarism is not uncommon but usually forgotten [2]. This can be also in concordant with any form of copyright violation (such as copying of figure or graph or equation or table) [3]. The good example is my discussed case in Account Res, “Singapore Ped Soc. 1978; page 122–141 in English and *Chula Med* *J*. 1980; the page in Thai language” [4]. Attempts to screen the translational plagiarism should be supported. The use of Google Translate tool might be helpful. Special focus should be on any non-English reference that might be the source of plagiarised material and non-English article that might translate from an original English article, which cannot be detected by simple plagiarism screening tool. It is a hard job for any journal to detect the complex translational plagiarism but the harder job might be how to effectively manage the case. Sometimes the case is reported to the affiliation but there is no action.
